# Inverse Regulation of the Cytosolic Ca^2+^ Buffer Parvalbumin and Mitochondrial Volume in Muscle Cells via SIRT1/PGC-1α Axis

**DOI:** 10.1371/journal.pone.0044837

**Published:** 2012-09-13

**Authors:** Sylvie Ducreux, Patrick Gregory, Beat Schwaller

**Affiliations:** Unit of Anatomy, Department of Medicine, University of Fribourg, Fribourg, Switzerland; University of Oldenburg, Germany

## Abstract

Skeletal muscles show a high plasticity to cope with various physiological demands. Different muscle types can be distinguished by the force, endurance, contraction/relaxation kinetics (fast-twitch vs. slow-twitch muscles), oxidative/glycolytic capacity, and also with respect to Ca^2+^-signaling components. Changes in Ca^2+^ signaling and associated Ca^2+^-dependent processes are thought to underlie the high adaptive capacity of muscle fibers. Here we investigated the consequences and the involved mechanisms caused by the ectopic expression of the Ca^2+^-binding protein parvalbumin (PV) in C2C12 myotubes *in vitro,* and conversely, the effects caused by its absence in in fast-twitch muscles of parvalbumin null-mutant (PV−/−) mice *in vivo*. The absence of PV in fast-twitch muscle *tibialis anterior* (TA) resulted in an increase in the peroxisome proliferator-activated receptor γ coactivator 1α (PGC-1α) and of its positive regulator, the deacetylase sirtuin 1 (SIRT1). TA muscles from PV−/− mice also have an increased mitochondrial volume. Mild ionophore treatment of control (PV-devoid) C2C12 myotubes causing a moderate elevation in [Ca^2+^]_c_ resulted in an increase in mitochondrial volume, together with elevated PGC-1α and SIRT1 expression levels, whilst it increased PV expression levels in myotubes stably transfected with PV. In PV-expressing myotubes the mitochondrial volume, PGC-1α and SIRT1 were significantly lower than in control C2C12 myotubes already at basal conditions and application of ionophore had no effect on either one. SIRT1 activation causes a down-regulation of PV in transfected myotubes, whilst SIRT1 inhibition has the opposite effect. We conclude that PV expression and mitochondrial volume in muscle cells are inversely regulated via a SIRT1/PGC-1α signaling axis.

## Introduction

Excitable cells, including muscle and nerve cells, need to cope with a vast range of activities, from low activity under basal or “resting” conditions to maximal activity occurring during brief, fast movements or complex cognitive processes, for example. This requires rapidly adapting systems, both for energy metabolism and for intracellular signaling and evidently the two need to be interconnected. Ca^2+^ ions serving as universal signaling molecules are strongly implicated in muscle function and Ca^2+^ signaling needs to be precisely orchestrated in space, amplitude and frequency in order to elicit the appropriate output signal. Cells make use of the Ca^2+^ signaling toolkit components [1] that allows transient alterations of the intracellular cytosolic Ca^2+^ concentration [Ca^2+^]_c_ in a highly coordinated way. One of these components present in the cytosol of fast-twitch muscles is parvalbumin (PV), a member of the large family of EF-hand Ca^2+^-binding proteins [2], [3] considered as a Ca^2+^ buffer or more precisely Ca^2+^ signal modulator [4]. For the proper functioning of a muscle cell, the intracellular Ca^2+^ signaling needs to be finely tuned and it is not surprising that disturbances in the Ca^2+^-signaling machinery (e.g. due to genetic mutations) affects the physiology of muscle cells and leads to various muscle disorders, e.g. muscular dystrophies [5]. The components of the Ca^2+^ signaling toolkit not only necessitate functioning in a concerted manner, but also have to be expressed dynamically in such a way as to fulfill the requirements of various demands posed by the physical activity of muscles. That is, besides the functional crosstalk between Ca^2+^ signaling components, there also exists a complex crosstalk at the level of transcription/translation and the signaling pathways involved in this process are slowly emerging [6], [7].

Besides the modulation of contractile properties of muscle, skeletal muscles demonstrate a high degree of plasticity in order to adapt to altered physiological demands resulting from increased exercise or caloric restriction, for example. A change in muscle fiber type from fast to slow or *vice versa* is not only accompanied by a switch in the expression of fiber-type specific filaments, in particular myosin heavy chain (MHC) isoforms, but those transitions also include shifting energy substrate (oxidative) metabolism and adaptation in mitochondrial biogenesis. The Ca^2+^-dependent mechanisms and the involved signaling pathways for the different processes are not entirely clear, but are likely to also involve Ca^2+^-dependent kinases (Ca^2+^/calmodulin-dependent kinases (CaMKII/IV), AMP-activated protein kinase (AMPK)) and phosphatases including calcineurin (CaN) [6], [8]. The peroxisome proliferator-activated receptor gamma coactivator-1 alpha (PGC-1α) has emerged as a decisive factor coordinating the activation of genes implicated in muscle metabolism including mitochondria biogenesis and fiber-type specificity [9]. In cultured skeletal muscle cells, PGC-1α is sufficient to activate transcription pathways in cooperation with myocyte enhancer factor (MEF2 proteins) that are linked to slow fiber (type I) gene expression [10]. In myotubes, sirtuin 1 (SIRT1) is the main deacetylase of PGC-1α that positively regulates genes in fatty acid metabolism and in mitochondrial respiration [11]. SIRT1 is capable of driving muscle-specific gene expression and metabolism involving MyoD, and SIRT1-dependent PGC-1α expression is crucial for this action [12]. PGC-1α complexes in neurons act as regulators of mitochondria density [13] and the PGC-1α/SIRT1 pathway has been suggested to act as a neuroprotective pathway [14], [15].

Elimination of PV in null-mutant mice (PV−/−) leads to cell-specific modifications/homeostatic adaptations that are not restricted to the level of other Ca^2+^-binding proteins. PV−/− fast-twitch muscles maintain their fast-twitch phenotype with respect to MHC isoforms, but the mitochondria content is significantly increased [16] and the mitochondria protein composition is as in slow-twitch muscle [17]. Thus, these mice serve as a unique model to dissect the multiple signaling pathways involved in the likely Ca^2+^ signaling-dependent mitochondrial biogenesis, while not affecting fiber-type specificity. Moreover, an inverse correlation between PV content and mitochondria volume is also seen in PV-expressing neurons including cerebellar Purkinje cells [18] or neurons with ectopic PV expression [19]. Here, we set out to investigate in greater detail, the mechanisms regulating PV expression and mitochondrial volume in two models: in C2C12 myotubes (PV-negative control cells and stably overexpressing PV-clones serving as a gain-of-function model) and in fast-twitch *tibialis anterior* (TA) muscles from wildtype (WT) and PV−/− mice serving as a loss-of-function model. An ionophore-mediated persistent elevation in [Ca^2+^]_c_ in PV-expressing myotubes increased PV expression and negligibly affected mitochondria biogenesis, whilst the rise in [Ca^2+^]_c_ upregulated PGC-1α and mitochondria volume in PV-negative control C2C12 myotubes. Both PGC-1α and its regulator SIRT1 were also increased in TA of PV−/− mice, along with the increased mitochondria volume in these muscle fibers described before [16]. PV expression and mitochondria volume in skeletal muscle are likely to be inversely regulated through a bidirectional regulation of the PGC-1α/SIRT1 signaling axis.

## Methods

### Ethics Statement

All experiments were performed in accordance with the guidelines of the European Committee Council Directive of November 24, 1986 (86/609/EEC) and the study was approved by the Veterinary Office of Fribourg, Switzerland.

### Materials

DMEM, penicillin, streptomycin, horse and fetal calf serum and G418 geneticin were purchased from GIBCO Invitrogen, Basel, Switzerland. Cell culture plasticware was from Milian AG, Geneva, Switzerland. Anti-parvalbumin and anti-calcineurin A antibodies were from Swant, Bellinzona, Switzerland, anti-SIRT1, anti–PGC-1α and ferutinin were from Santa Cruz Biotechnology Inc., Santa Cruz, CA, U.S.A. Sirtinol, resveratrol were from Sigma. Calcein AM, rhodamin 123, rhod-2 AM, Fura-2 AM, MitoTracker Green FM, MitoTracker Red CMXRos, Hoechst and 4-bromo A23187 calcium ionophore were purchased from Molecular Probes BV, Leiden, The Netherlands. Glass coverslips were from Assistent. JetPEI™ transfection reagent was from Brunschwig, Basel, Switzerland. All other chemicals were of highest available grade.

### Animals

PV-deficient mice (PV−/−) were generated on a C57Bl/6J x 129S background by homologous recombination [20] and backcrossed to C57Bl/6J for 10 generations and are thus considered to be congenic with wild type (WT) C57Bl/6J [21]. All experiments were performed using 2- to 6-months-old adult male mice of both genotypes (PV−/− and WT). Mice were deeply anesthetized by the inhalation of CO_2_ and perfused transcardially by ice-cold saline solution (0.9%) or phosphate-buffered saline (PBS). Muscles were immediately dissected, snap frozen in liquid nitrogen and stored at −70°C until analysis. Tissue used for RNA isolation was dissected from mice perfused with sterile ice-cold PBS prepared with diethyl pyrocarbonate (DEPC)-treated water.

### Cell Culture

C2C12 mouse myoblast cells were obtained from ATCC (Rockville, MD). C2C12 cells were routinely cultured at 37°C in a humidified atmosphere of 95% air and 5% CO_2_ in growth medium (GM): Dulbecco’s modified Eagle’s medium (DMEM) supplemented with 10% heat-inactivated fetal calf serum (FCS), 100 units/ml penicillin, 100 µg/ml streptomycin. Cells were passaged by trypsinization every 2–3 days when approximately 60–70% confluence was reached. Differentiation into myotubes was induced using differentiation medium (DM), i.e. DMEM supplemented with 2% horse serum (HS) for 6–10 days.

### Stable Transfection with Parvalbumin Expression and GFP Expression Plasmids

The transfection procedure was performed as described before [22]. The expression plasmid RSV-PV-NEO contains the RSV promoter, the 3′ untranslated region and poly-(A) signal for rat preproinsulin, a short linker region for the insertion of the rat PV cDNA comprising the entire open reading frame of PV and a neomycine resistance cassette [23]. The plasmid was selected based on its previous use in other studies [22] and moreover, Ca^2+^-binding properties of rat and mouse PV are almost identical, also based on the fact that the two Ca^2+^-chelating loops are 100% identical in rat and mouse PV and the overall identity at the amino acid level is 95%. The EGFP-C1 expression plasmid was obtained from Clontech (Mountain View, CA). Myoblasts were seeded into 12-well plates at a concentration of 10^4^ cells/well. Stable transfection was performed by the use of the JetPEI™ transfection reagent and a total of 2 µg linearized plasmid DNA per well. The day after transfection, the medium was changed to fresh GM. After an additional 24 h, cells were incubated with fresh GM supplemented by geneticin (G418, 500 µg/ml, Life Technologies, Basel, Switzerland) for 10–12 days. Isolation of individual transfected G418-positive clones was obtained by serial dilution.

### Protein Extraction

Isolated muscles or C2C12 cell samples were sonicated in homogenization buffer (10 mM Tris–HCl, 1 mM EDTA, pH 7.4, containing a protease inhibitor cocktail; Roche, Rotkreuz, Switzerland). After centrifugation at 13,000 rpm for 5 min on a bench top centrifuge, the supernatant was retained for analysis of cytosolic proteins. To generate fractions enriched in nuclear proteins, samples were prepared as previously described [24]. Briefly, 2×10^6^ cells were harvested and washed in PBS. Thawed muscles and harvested C2C12 cells were homogenized in ice-cold buffer 1 (10 mM HEPES pH 7.5, 10 mM MgCl_2_, 5 mM KCl, 0.1 mM EDTA, pH 8.0, 0.1% TritonX-100, 1 mM DTT, 0.1 mM PMSF, 2 µg/ml aprotinin and 2 µg/ml leupeptin) and centrifuged at 3000×g for 5 min at 4°C. Pellets were then resuspended in ice-cold buffer 2 (20 mM HEPES pH 7.9, 25% glycerol, 500 mM NaCl, 1.5 mM MgCl_2_, 0.2 mM EDTA, pH 8.0, 0.5 mM DTT, 0.2 mM PMSF, 2 µg/ml aprotinin and 2 µg/ml leupeptin), incubated 30 min on ice with occasional mixing and centrifuged at 3000×g for 5 min at 4°C. Supernatants from muscles or C2C12 cells were transferred to Amicon Ultra 5000 NMWL, 4 ml Ultrafree Filter Unit or Ultra 0.5 30 K centrifugal devices (Millipore), respectively and centrifuged at 7000×g for 1 h or 30 min at 14,000×g at 4°C in order to concentrate the solutes. Total protein concentration in all extracts was determined using the Bradford or D_c_ assays (Pierce, Thermo Scientific, USA).

### Immunohistochemistry and Western Blot Analysis

Differentiated C2C12 myotubes were fixed with 4% PFA and immunostained with an antiserum against PV, using the polyclonal antiserum PV25 (1∶1000; Swant, Bellinzona, Switzerland) and Cy3-labeled donkey anti-rabbit secondary antibody (1∶1000; Vector Laboratories, Burlingame, CA, USA). For Western blot analyses, proteins (20 µg or 50 µg) were separated on either 7.5% or 12.5% polyacrylamide gels (depending on protein of interest) and transferred onto nitrocellulose membranes using a semidry transfer protocol. After blocking with 5% solution of non-fat milk in TBS buffer, membranes were incubated with the relevant primary antibodies. Two types of secondary antibodies were used: either Li-Cor® infrared dye-labeled antibodies or biotinylated antibodies (1∶10,000; Vector) accompanied with additional incubation with avidin–biotin-conjugated peroxidase solution (Vector). When the secondary antibodies were Li-Cor® antibodies, all antibodies were diluted in the Li-Cor® blocking reagent and bands were detected using the Li-Cor™ Odyssey system. All image analyses were performed with the free software ImageJ (NIH). All membranes were stained with Ponceau S to confirm equal loading and to enable normalization for purposes of densitometry. The integral of all proteins was used in order to prevent a bias from any single reference or house-keeping protein.

### RNA Extraction and Reverse-transcription PCR

Total RNA was extracted from samples using the Trizol reagent according to the manufacturer’s protocol. 2 µg of total RNA was subsequently reverse-transcribed using the RevertAid™ H Minus First Strand cDNA Synthesis Kit, again following the manufacturer’s instructions. An aliquot from this reaction, equivalent to 200 ng of un-transcribed RNA, was amplified with GoTaq™ Flexi DNA polymerase using conditions recommended by the manufacturer. Reactions were performed on an MJ Research PCR machine. Details of oligonucleotides sequences are shown in Table 1.

**Table 1:** PCR primer sequences used for RT-PCR.

Gene	Forward	Reverse
AMPK	CCACGAGAGCCTAGGTGAAG	TTCCAAGATCCTTTCGTTGG
CaN	AGTAACAATTTTCAGTGCTCCAAAC	1) AATATACGGTTCATGGCAATACTGT2) ACTTAAGAGTGTCGTCAAGTTCCAT
CaMKIV	TCCTCTGGGCGATTTCTTCG	CTGATTTCTGTGGGGGTTTCG
CaMKIIδ	CTACCCCGGCGCTGGAGTCAAC	1) TCAGATGTTTTGCCACAAAGAGGTGCCTCCT2) TGCTTTCGTGCTTTCACGTCTTCA
GAPDH	GAGCTGAACGGGAAGCTCACTGG	CAACTGTGAGGAGGGGAGATTCAG
PGC-1α	1) AGTGTGCTGCTCTGGTTGGTG2) CGAAGAGCATTTGTCAACAGCA	GGAGGGTCATCGTTTGTGGTC
PKCα	CCCATTCCAGAAGGAGATGA	TTCCTGTCAGCAAGCATCACT
PV	TCCAGATGGTGGGGCCTGAAGAAAAAGAGTG	GTCCCCGTCCTTGTCTCCAGCAGCCATC

In order to increase the reliability of PCR results, for certain genes 2 sets of primer pairs were used, either 2 different reverse primers (CaN, CaMKIIδ) or forward primers (PGC-1α).

### Flow Cytometry

Control C2C12 cells and PV-clones were incubated in basal media or media containing the Ca^2+^ ionophore Br-A23187 (1 µM). Cells were trypsinized for 5 min and DMEM medium was added to stop the reaction. Cells were centrifuged at 1,000 rpm for 5 min, resuspended and counted. In order to assess the cellular mitochondrial mass, cells were stained with MitoTracker Green FM (200 nM), a mitochondrial specific fluorescent dye at 37°C for 30 min. After incubation, cells were washed and resuspended in PBS at a concentration of 0.5×10^6^ cells/ml. Cell counts were performed on a FACS CyFlow space (Partec GmbH, Germany) using FloMax® software. The forward scatter vs. side scatter area was used to analyze a homogeneous population of live cells and to determine FACS size gating. One part of C2C12 cells was processed without MitoTracker Green incubation and was used as a negative control. FACS quantitative and statistical analyses were performed with the FlowJo software (Tree Star, Inc. Ashland, OR).

### 3D Measurements

C2C12 cells were transferred onto glass coverslips (4.2 cm diameter) and allowed to attach, proliferate in growth medium and then in differentiation medium. Calcein-AM (green), DAPI (blue) and Mito Tracker Red (red) signals were acquired with a Leica TCS SP5/DMI6000 confocal microscope using a 20x water-immersion HCX PL APO objective (0.70 numerical aperture). Z-stacks were composed of 30–50 adjacent sections, of optical slices of 400–800 nm step size. Images were obtained from control and PV-expressing C2C12 cells at day 5 in differentiation medium (n>30 cells). For these measurements, only cells with a single nucleus and not multi-nucleated cells were analyzed. For measurements of the whole cell and nuclear volumes, complete series of z-stacks were processed using Imaris software (Bitplane AG, Switzerland) with the “isosurface” routine using automatic fluorescent threshold from the green and blue signal, respectively. The mitochondrial volume was determined from the detecting “spots” procedure by applying an identical intensity threshold to the red channel.

### Single Cell Measurements

C2C12 cells were grown and differentiated on glass coverslips. For measurements, coverslips were mounted onto a 37°C thermostatted chamber, which was continuously perfused with Krebs Ringer medium; individual cells were stimulated with the aid of an 8-channels computer-controlled multifunction superfusion system (OctaFlow 08S, ALA Scientific Instruments, Inc. Westbury, NY). On-line measurements were recorded using a confocal Leica DMI6000 inverted fluorescent microscope equipped with UV and visible laser lines. To investigate changes in mitochondrial membrane potential and in [Ca^2+^]_m_, cells were loaded with the AM ester of rhodamin 123 and rhod-2 (5 µM; *K*_d_ = 570 nM) at 37°C for 30 min in culture medium, followed by a subsequent 30 min period in dye-free medium for hydrolysis of the dye. The positively charged rhodamin 123 preferentially accumulates in the mitochondria due to the negative mitochondrial membrane potential of 150–180 mV and its fluorescence is quenched inside these organelles. Upon intramitochondrial Ca^2+^ increase, Δψ_m_ collapses, rhodamin 123 is transported back to the cytoplasm and thus the fluorescence (F/F_0_) increases. Loaded cells were excited at 488 nm and 543 nm at a laser power of 20 and 25%, respectively, scan speed was at 8000 Hz and the pinhole was set at 1 Airy unit using a 20x water-immersion HCX PL APO objective (0.70 numerical aperture). Rhodamin 123 and rhod-2 fluorescence emissions were simultaneously captured by 2 PMTs at 509–555 nm and 587–677 nm emission ranges, respectively. To measure cytosolic [Ca^2+^]_c_ dynamics, cells were loaded with Fura-2 AM (5 µM) in Krebs Ringer (KR) medium for 30 min at 37°C. Prior to experiments, cells were rinsed twice with Ca^2+^-free KR solution to remove non-hydrolyzed indicator. Imaging was acquired with a digital cooled CCD camera (Photometrics, USA) on an inverted stage microscope (Nikon Diaphot) using a 40x oil-immersion objective. Fura-2 was excited alternatively at 340 and 380 nm using a dual monochromator (Polychrome II, Photonics, Germany), emission fluorescence was selected through a dichroic mirror (DM400) and a wide-band filter (520–560 nm). Ca^2+^ signals were analyzed using the Metafluor software (Universal Imaging Corporation®, West Chester, PA). Regions of interest (ROIs) were defined within individual cells. After background subtraction, the ratio F_340nm_/F_380nm_ was calculated to estimate intracellular [Ca^2+^].

### Data Evaluation and Statistical Analysis

Analysis of the live *in situ* imaging data was performed using the LAS-AF program (Leica). For the calculation of rhodamin123 and rhod-2 fluorescence intensity ratios, the following procedure was used: the fluorescent cells were outlined as regions of interest (ROI); the mean grey-scale values of the ROIs were calculated in both rhodamin123 and rhod-2 image sets. The obtained values were extracted as.csv files and transferred to OriginPro 8.1 Pro (OriginLab Corporation, Northampton, MA, USA), where all subsequent calculations and graphs were performed. Ratio plots were calibrated relative to the basal (pre-stimulus) normalized fluorescence; the average values used for normalization considered only images after a stable base line of dye ratios were reached. The kinetics of mitochondria signals were determined by the fluorescence intensities of entire cell-shape ROIs on the respective X-Y-t records. Other data were calculated using the mean and SEM. Significance was determined with Student’s *t* test.

## Results

### Generation and Characterization of C2C12 Myotubes with Ectopic PV Expression

**Figure 1:**
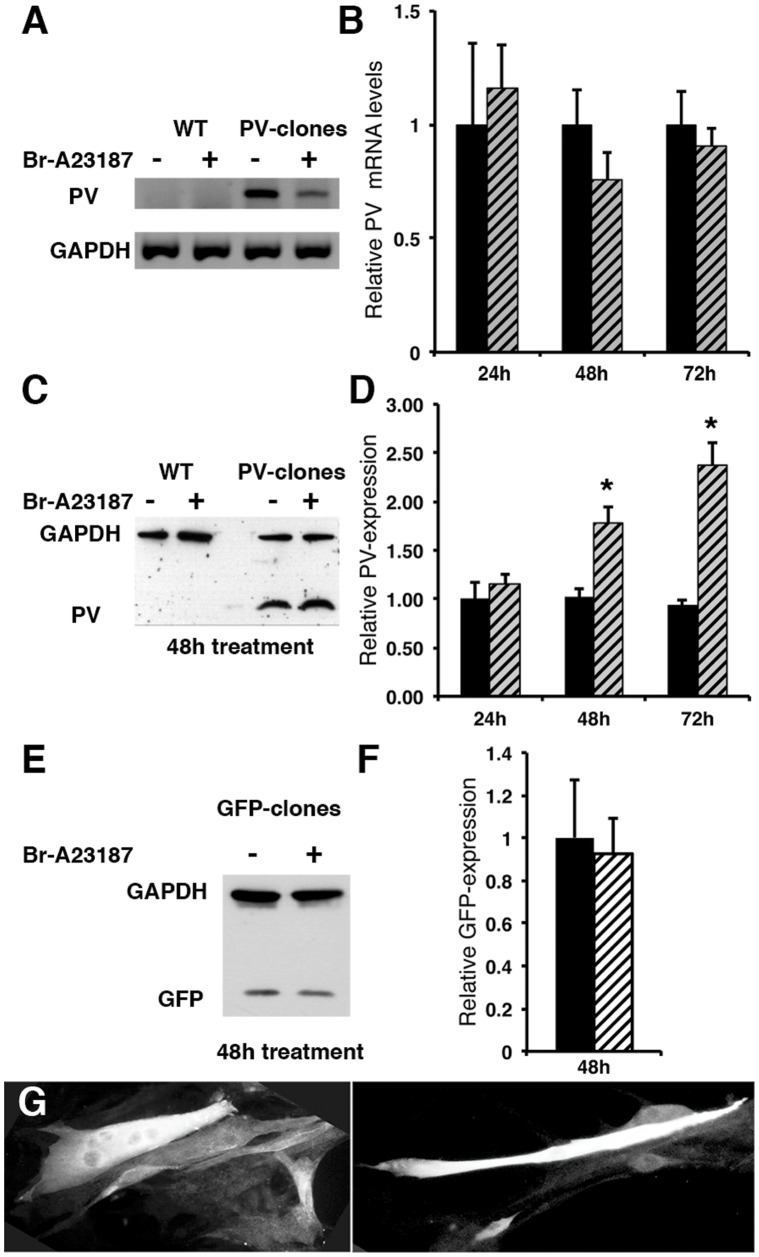
Effect of mild ionophore treatment on PV expression levels in PV-transfected C2C12 cells. A) Detection of mRNA for PV and GAPDH (for normalization) in control (WT) and PV-transfected myotubes by RT-PCR. No signal was detected in WT myotubes before and after ionophore treatment. B) Ionophore treatment did not significantly affect PV mRNA levels (striped bars) of PV-clones at 24–72 h after treatment in comparison to untreated cells (black bars). C) Protein expression levels of PV and GAPDH in WT and PV-clones subjected to Ca^2+^ ionophore treatment (1 µM Br-A23187 for 48 h (+) or untreated cells (−) determined by Western blot analysis. D) Semi-quantitative analysis of PV Western blot signals in C2C12 PV-clones exposed to Br-A23187 for 24–72 h (striped bars) in comparison to untreated cells (black bars). A significant increase in PV expression levels was observed at 48 and 72 h (*; p<0.05; n = 8 values at each time point). E–F: Relative GFP protein expression in GFP-positive C2C12 clones in total protein extracts. Cells were treated for 48 h with the differentiation medium alone (black bar) or with 1 µM Br-A23187 (striped bar). A representative Western blot from clone PV1 is shown in E. For the normalization, GAPDH and/or the Ponceau Red-staining of the nitrocellulose membrane were used. Values were from 2 or 3 clones (PV1–3) and ≥2 independent experiments/clone. G: Stably-transfected C2C12 cells differentiated to myotubes for 6 days were fixed and immunostained for PV expression. PV in multinucleated myotubes is homogenously expressed throughout the cytoplasm.

An inverse correlation exists between PV expression levels and mitochondrial volume in fast-twitch muscle fibers [16] and neurons [18], [19]. Here, we investigated the mechanistic/molecular details of the regulation in a model system, using C2C12 myotubes. With respect to MHC isoforms, C2C12 cells consist of a mixed cell population expressing type I or type II (IIa, IIx or IIb) isoforms [25], [26]. Since PV is not expressed in mammalian type I fibers and PV expression is rapidly lost also in type II fibers cultured *in vitro*, we verified that C2C12 control myotubes (WT) are devoid of PV expression. No expression of PV mRNA transcripts or protein was found in control myotubes (Fig. 1A, C). Thus they represent a slow-twitch phenotype with respect to PV expression. We then generated PV-expressing clones by stably transfecting C2C12 myoblasts with an expression plasmid coding for full-length PV. In transfected myotubes (PV-clones), PV mRNA (Fig. 1A) and PV protein (M_r_: 12 kDa) evidenced by Western blot (Fig. 1C) were clearly visible as shown for a representative clone PV1. This clone was selected due to its high PV expression level and, as the other clones (PV2 & PV3), based on unaltered cell proliferation and morphology compared to control C2C12 cells. Clones with slightly lower PV expression levels were tested in various experiments and yielded essentially identical results as with clones PV1-3, however effects were generally smaller (data not shown). In addition, the intracellular distribution of PV in the PV-clones was analyzed by immunofluorescence. PV-immunoreactivity in multi-nucleated myotubes was present throughout the cytoplasm and was weaker in nuclear regions (Fig. 1G). Hence, a comparison of C2C12 myotubes with or without expression of PV serves as a valuable gain-of-function model to investigate the function of PV and its effects on mitochondria biogenesis.

### Effect of the Calcium Ionophore Br-A23187 on PV Expression in C2C12 Myotubes

Calcium ionophore exposure elevates both cytosolic [Ca^2+^]_c_ and intramitochondrial [Ca^2+^]_m_ in cultured cells [27], [28]. In order to investigate whether PV expression levels (mRNA and protein) were affected by increasing [Ca^2+^]_c_ using a relatively low concentration (1 µM) of Br-A23187, PV-transfected and control myotubes were exposed to Br-A21387 for 24, 48 and 72 h. This concentration of Br-A23187 was previously shown not to induce cytotoxicity in cultured newborn-rabbit derived primary muscle cells [29] and myotubes [30]. C2C12 cell survival was estimated by calcein-AM loading, cells displayed similar fluorescence distribution and staining intensity in ionophore-treated or untreated myotubes up to 48 h treatment, indicating that C2C12 myotubes with or without PV expression support the ionophore-mediated increase in [Ca^2+^]_i_ (see below). Ionophore treatment increased PV protein expression levels in PV-clones in a time-dependent manner; a 1.77-fold±0.17 (p<0.05) increase at 48 h; results are the average from at least 2 control and 2 PV-transfected clones and protein extracts were isolated from at least 2 independent experiments (Fig. 1D). The maximal increase (237%±25% of control) was reached after 72 h of Br-A23187 treatment. In order to exclude that ionophore treatment leads to an unspecific upregulation of ectopically expressed proteins in C2C12 myotubes, clones stably transfected with the plasmid pEGFP-C1 encoding the enhanced green fluorescent protein (EGFP) were generated and analyzed (Fig. 1E). Treatment of these cells with ionophore for 48 h did not enhance EGFP expression in comparison to untreated cells (ratio ionophore-treated/control: 0.92±0.16; Fig. 1F), demonstrating the specificity of the effect for PV. At the level of mRNA, results from semi-quantitative RT-PCR indicated no significant increase in PV mRNA in the PV-transfected, ionophore-treated cells (Fig. 1B). Of importance, in transfected cells, PV expression is not driven by the endogenous *Pvalb* promoter, but by the viral RSV promoter. The finding that PV protein, but not mRNA levels was elevated after ionophore treatment indicates a post-transcriptional mechanism. Thus, elevated [Ca^2+^]_c_ increases PV protein levels independent of the activity of the *Pvalb* promoter, indicative of yet another mechanism than those mediated by Ca^2+^-responsive promoter elements [31], [32] or with an involvement of Ca^2+^-modulated transcription factors (e.g. bHLH [33]). The next step was to investigate the effects of ionophore treatment on the mitochondria of PV-transfected and control C2C12 cells.

### Impact of Ionophore-induced Persistently Increased Levels of [Ca^2+^]_c_ on the Mitochondrial Content of C2C12 Cells

**Figure 2:**
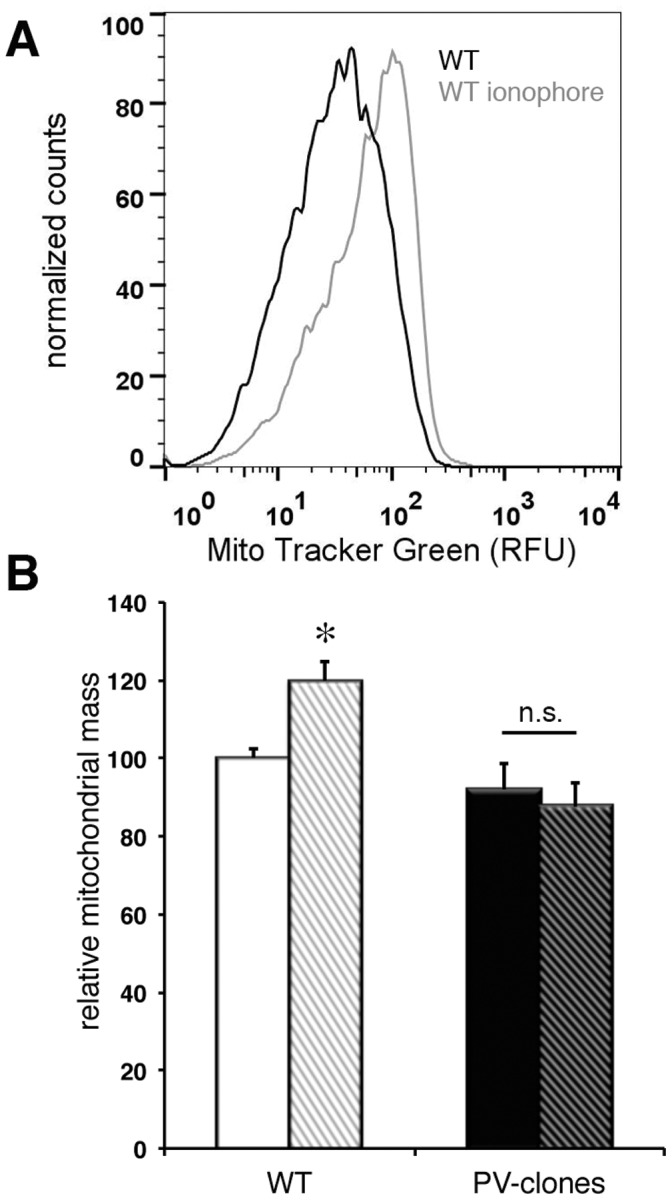
Increased mitochondrial mass determined by FACS analysis in control (WT) C2C12 clones exposed to Br-A23187. A) FACS histogram of WT C2C12 cells treated for 48 h with Br-A23187 (1 µM) and loaded with Mito Tracker Green. A shift to the right (increased relative fluorescence units (RFU)) was observed in WT C2C12 cells. B) Quantitative analysis of relative mitochondrial mass in WT (light bars) and PV-clones (dark bars), before (left bars) and after ionophore treatment (right bars). After ionophore treatment, the mitochondrial mass was significantly increased in the WT clones (*; p<0.05). In ionophore-treated PV-clones, the relative mitochondrial mass was essentially unchanged, the small decrease was not significant (n.s.). In untreated WT and PV-clones, the small difference was also not significant. Each FACS count was of 20,000 particles (n ≥4 experiments).

**Figure 3:**
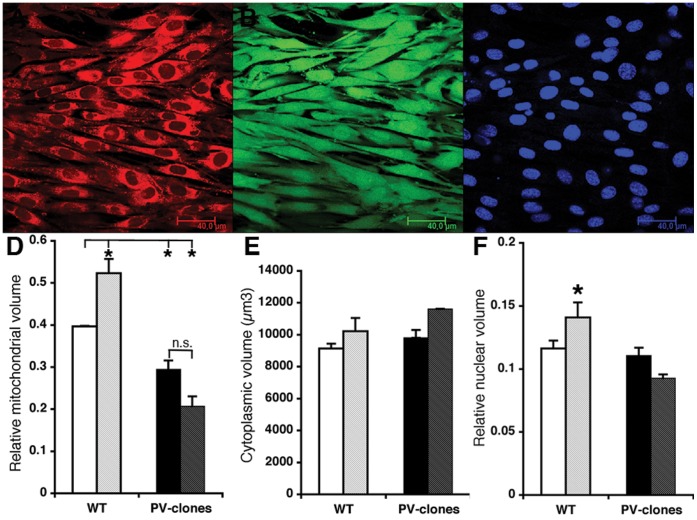
Volumetric analysis of mitochondrial content in WT and PV-transfected C2C12 cells. A–C: Confocal images of WT C2C12 cell loaded with Mito Tracker Red (A), Calcein-AM (B), DAPI (C): x–y image Z-stack projection (large image), x–z (bottom) and y-z (right) image. Scale bar is 40 µm. These images were transferred to Imaris 3D to calculate relative mitochondrial volume. D–F: Bar histographs of relative mitochondrial volume (D), cytoplasmic volume (E) and relative nuclear volume (F) in C2C12 WT (light bars) and PV-clones (dark bars) either in control medium (left bars) or incubated for 48 h with Br-A23187 (1 µM; right bars). D) The relative mitochondrial volume was increased in WT C2C12 after ionophore treatment (*; p<0.05). The mitochondrial volume in PV-clones, both in control medium or after 48 h ionophore treatment was lower than in untreated WT C2C12 cells; * p<0.05 vs. WT; n>30 cells). The decrease in mitochondrial volume in PV-clones after ionophore treatment was not significant (n.s.) E) No differences in cytoplasmic volumes were detected. F) The nuclear volume in ionophore-treated WT C2C12 was slightly larger than in untreated WT cells (*; p<0.05).

The relative mitochondrial volume in fast-twitch muscles (and cerebellar Purkinje cells) of PV knockout (PV−/−) mice is increased [16], while it is decreased in neurons ectopically expressing PV [19]. Thus, we expected to get insight in the putative molecular mechanism regulating PV expression and mitochondrial volume in an antagonistic way using the C2C12 model system. The relative mitochondrial mass (volume) was determined by two complementary methods, with the aim of obtaining reliable results on the mitochondrial volume regulation in C2C12 cells. The total mitochondrial mass was first assessed by fluorescent-activated cell sorting (FACS; Fig. 2A). After 6–10 days incubation in differentiation medium, C2C12 myotubes were stained with MitoTracker Green, a specific dye accumulating in mitochondria regardless of mitochondrial membrane potential. In PV-transfected myotubes the mitochondrial mass was slightly, however not significantly, reduced when compared to non-transfected control myotubes, already when grown in basal media (Fig. 2B). Mild ionophore treatment was used as a proxy process mimicking muscle activity. The right shift in the FACS histogram of control cells treated with Br-A23187 (1 µM) for 48 h indicated an increase in mitochondrial mass; quantitative analysis revealed the increase to be 20±5% (p<0.05; Fig. 2B). The mitochondrial mass of ionophore-treated PV-clones was not different from control PV-clones. Since data were acquired by size-gating analysis, the increase in MitoTracker fluorescence precluded an increased cell size to be the cause of the larger FACS signals in ionophore treated myotubes. As a complementary approach, 3D-reconstruction analysis from cell cultures *in vitro* was performed in cell clusters comprising 3–10 cells. Cells were stained with MitoTracker Red CMXRos (mitochondria; Fig. 3A), calcein-AM (cytosol; Fig. 3B) and Hoechst 33342 (nuclei; Fig. 3C). Visual inspection at higher magnifications revealed “normal” mitochondria morphology as seen before in fast-twitch muscles and Purkinje cells of PV−/− mice [16], [18], i.e. no signs of swelling (rounding) characteristic for mitochondrial Ca^2+^ overload. 3D-surface volume renderings of the total mitochondrial population, the total cytoplasmic volume and the nuclear volume were carried out on PV-transfected and control cells exposed to Br-A23187 for 48 h and an example of control C2C12 cells is depicted in Fig. 3. As a first observation, the distribution of mitochondria within the cells was not affected by the ionophore treatment; mitochondria remained scattered throughout the cell, most often enriched in a perinuclear region in both, PV-expressing and control cells. The cytoplasmic volume was not different in the two types of C2C12 clones, neither before nor after ionophore treatment (Fig. 3E). The volume of nuclei was slightly increased in ionophore-treated WT C2C12 in comparison to control C2C12 cells (Fig. 3F). The relative mitochondrial volume in PV-expressing cells, i.e. the relative volume labeled with MitoTracker in the cytoplasmic compartment, was reduced by approximately 10%, from 40±1% (in WT) to 29±2% in PV-clones; p<0.05 (Fig. 3D). Addition of ionophore to control cells for 48 h increased the relative mitochondrial volume from 40±1% to 52±3% (p<0.05), whereas it remained essentially unchanged in PV-transfected cells; the slight decrease was not significant (Fig. 3D; p = 0.13). Also when the mitochondrial volume was normalized to the cytoplasmic volume of each cell, the significant differences persisted between the groups. Thus, the morphometry results indicated a significant increase in mitochondrial volume induced by the ionophore treatment selectively in cells not expressing PV. In PV-clones, PV protein levels were upregulated instead (Fig. 1C, D).

### Differences in the Kinetics of Mitochondrial Ca^2+^ Sequestration in PV-expressing C2C12 Myotubes

Due to PV’s Ca^2+^-binding properties and high expression in rodent fast-twitch muscles, PV participates in shaping intracellular Ca^2+^ signals by sequentially acting as a Ca^2+^ sink (buffering phase) and a Ca^2+^ source (release phase) during each contraction-relaxation cycle. That mitochondria also behave in a similar way in skeletal muscle is a more recent finding [34]. Based on the rather similar kinetic properties of mitochondria and PV, i.e. a delayed Ca^2+^-buffering/uptake that only mildly affects the rising phase of [Ca^2+^]_c_ transients, it was proposed that mitochondria might represent a valuable substitute for PV in fast-twitch muscles of PV−/− mice. Exposure of C2C12 myotubes to increased [Ca^2+^]_c_ could hypothetically lead to adaptive mechanisms to increase the intracellular Ca^2+^-buffering capacity; in control cells, by increasing the mitochondrial mass/volume (Figs. 2 & 3) and in PV-transfected cells by the upregulation of PV expression (Fig. 1C, D). Results presented above relied on ionophore treatment, which caused a moderate increase in [Ca^2+^]_c_.

**Figure 4:**
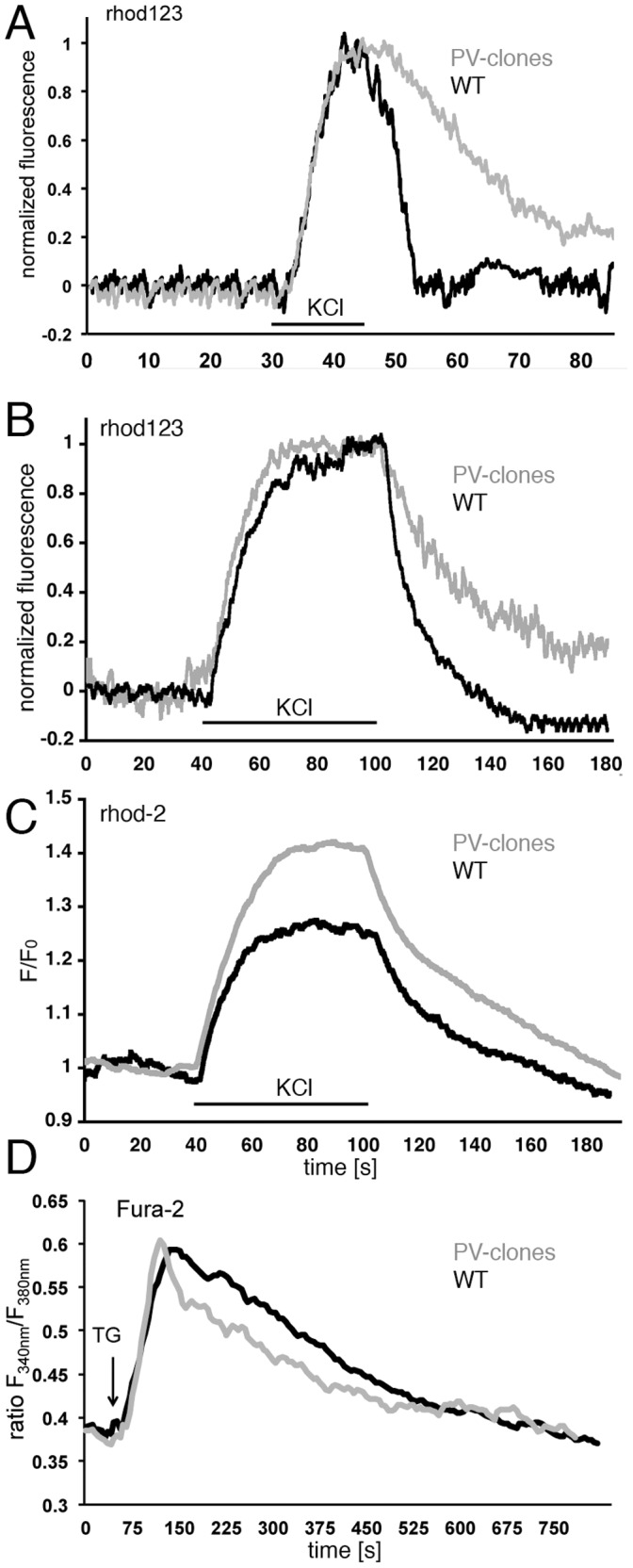
Effect of KCl-induced depolarization and thapsigargin treatment in C2C12 WT and PV-clones on mitochondrial membrane potential, [Ca^2+^]_m_ and **[Ca^2+^]_c_.** A) Representative (normalized) traces of rhod123-loaded C2C12 cells (WT, black line and PV-clone, gray line) exposed to nominally 100 mM KCl for 15 s starting at 30 s. Higher values indicate a decrease in intramitochondrial membrane potential (Δψ_m_). The average basal fluorescence before treatment (0–20 s) was subtracted. B) A similar experiment with cells exposed to nominally 300 mM KCl for 60 s starting at 40 s. The actual KCl concentration sensed by the cells is estimated to be less than half of the nominal concentration due to dilution with basal medium. C) Representative traces of rhod-2-loaded C2C12 cells (WT, black line and PV-clone, gray line) exposed to nominally 300 mM KCl for 60 s starting at 40 s. Higher values indicate an increase in [Ca^2+^]_m_. Changes in fluorescence are reported as F/F_0_, where F_0_ is the baseline reached before drug application. Quantitative data is presented in Table 2. D) WT and PV-clones were treated with thapsigargin (1 µM) and [Ca^2+^]_c_ was recorded with Fura-2; representative traces are shown, quantitative data (n = 9 cells, both from WT and PV-clones from 3 independent experiments) is presented in the Results section. Note the different time scales in A, B&C and D.

In the next series of experiments, the mitochondria’s capacity to cope with a rapid increase in [Ca^2+^]_c_ (i.e. by strong depolarization) was investigated. In C2C12 myotubes, changes in the mitochondrial membrane potential (ΔΨ_m_) and in the mitochondrial Ca^2+^ concentration [Ca^2+^]_m_ were measured by loading cells with the fluorescent dyes rhodamin 123 and rhod-2, respectively. As a paradigm to transiently elevate [Ca^2+^]_c_, cells were depolarized by local delivery of KCl-enriched buffer (concentration: 100 mM for 15 s and 300 mM for 60 s). The KCl-enriched buffers were applied via a thin glass pipette (flow: 0.1 ml/min) placed in the vicinity of the cells that were constantly perfused with KR solution (1.5 ml/min), thus the actual KCl concentration sensed by the cells was lower than the nominal concentration of applied KCl solutions, we estimated a dilution of at least 2-fold. The decrease in ΔΨ_m_ is reflected in the increase of the rhodamin 123 fluorescence (F/F_0_; Fig. 4A,B). After either exposure to nominally 100 mM KCl for 15 s (Fig. 4A) or nominally 300 mM KCl for 60 s (Fig. 4B), the medium was changed to basal media and rhodamin 123 fluorescence quickly recovered to pre-stimulation values, indicative of the reestablishment of ΔΨ_m_. Different parameters including rise time (10–90%) and the half-recovery time (τ_1/2_) were determined (Table 2). The shorter rise time observed in the PV-expressing clones (Table 2) that was more evident at the higher KCl concentration (Fig. 4B) indicated that the decrease in ΔΨ_m_ occurred faster than in the control myotubes characterized by a higher mitochondrial volume. The myotubes’ capacity to restore ΔΨ_m_ after removal of KCl was also clearly correlated with their mitochondrial volume/mass: repolarization was significantly faster (approximately −40% at nominally 100 and 300 mM KCl) in control myotubes than in those expressing PV (Table 2). Thus, mitochondria in myotubes clearly contribute to transient Ca^2+^ buffering/storage. Moreover, control C2C12 myotubes with increased mitochondrial mass/volume, are better able to resist a Ca^2+^-induced decrease/collapse in ΔΨ_m_ and can repolarize faster. A similar situation was also observed with respect to [Ca^2+^]_m_. The rise in mitochondrial [Ca^2+^]_m_ was faster at both KCl concentrations, ΔF/F_0, max_ at nominally 300 mM KCl was higher and τ_1/2_ reflecting the time required for restoration of basal [Ca^2+^]_m_ was longer in the PV-clones compared to control C2C12 cells (Fig. 4C and Table 2). In conclusion, the higher mitochondrial volume in control C2C12 myotubes when compared with PV-expressing myotubes resulted in improved Ca^2+^ handling/sequestering by mitochondria.

**Table 2:** Quantitative analysis of the kinetics of intramitochondrial membrane potential (ΔΨ_m_) and mitochondrial [Ca^2+^]_m_ changes in C2C12 WT and PV-clones after KCl-induced depolarization.

	100 mM# KCl	100 mM# KCl	300 mM# KCl	300 mM# KCl
	WT	PV-clones	WT	PV-clones
**Δψ_m_** (rhod123)				
*Rise time 10–90% (s)*	6.265±0.564	5.562±0.589	19.935±2.275	16.678±1.666
*½ recovery (s)*	6.962±0.825	13.102±1.494*	14.928±0.909	23.053±2.757**
**[Ca^2+^]_m_** (rhod-2)				
*Rise time 10–90% (s)*	6.669±1.367	3.778±0.456*	19.105±2.16	14.584±2.113*
*ΔF max*	0.346±0.137	0.336±0.111	0.278±0.021	0.397±0.028**
*½ decay (s)*	7.862±1.001	10.995±1.726*	14.926±1.125	31.224±3.413**

Results are from at least 3 independent myotube preparations and number of cells analyzed ranged from 6–16 per genotype (WT and PV-clones) and conditions (100^#^ and 300^#^ mM KCl; *p<0.05, **p<0.01).

# The concentrations represent values of the added solutions to the perfusion chamber. The actual concentration sensed by the cells is assumed to be at least 2-fold lower.

In order to exclude that the observed effects were due to a generally altered cytosolic Ca^2+^ handling in PV-clones, we ascertained that basic aspects of cytosolic [Ca^2+^]_c_ transients were not substantially altered in the presence of PV. Control C2C12 and PV-clones were exposed to thapsigargin (1 µM) to deplete ER/SR stores and the kinetics and amplitude of cytosolic [Ca^2+^]_c_ transients were evidenced by Fura-2 loading of cells. The ratio F_340nm_/F_380nm_ before stimulation was identical in WT and PV-clones (0.38±0.022 vs. 0.379±0.16, respectively) confirming that the presence of a Ca^2+^ buffer didn’t affect basal [Ca^2+^]_c_ (Fig. 4D). Also peak [Ca^2+^]_c_ induced by thapsigargin application was not significantly different (0.612±0.052 in WT vs. 0.606±0.037) in PV-clones. Small differences in the shape of [Ca^2+^]_c_ transients were discernable (Fig. 4D). Generally, the rate of rise in [Ca^2+^]_c_ was slightly faster in PV-clones, but on the other hand, the rate in [Ca^2+^]_c_ decay -evidenced by a shorter half-decay time- was faster in PV-clones. The delay to reach peak [Ca^2+^]_c_ in WT cells was correlated with their increased mitochondrial volume suggesting that mitochondria participate in Ca^2+^ removal/uptake already during the thapsigargin-induced rise in [Ca^2+^]_c_. On the other hand, the more rapid decay in [Ca^2+^]_c_ in PV-clones supports the role of Ca^2+^ buffers acting as shuttles and thus accelerating the removal of intracellular Ca^2+^ (for more details, see [3]).

### Signaling Pathways Involved in Mitochondria Biogenesis in C2C12 Cells *in situ*

**Figure 5:**
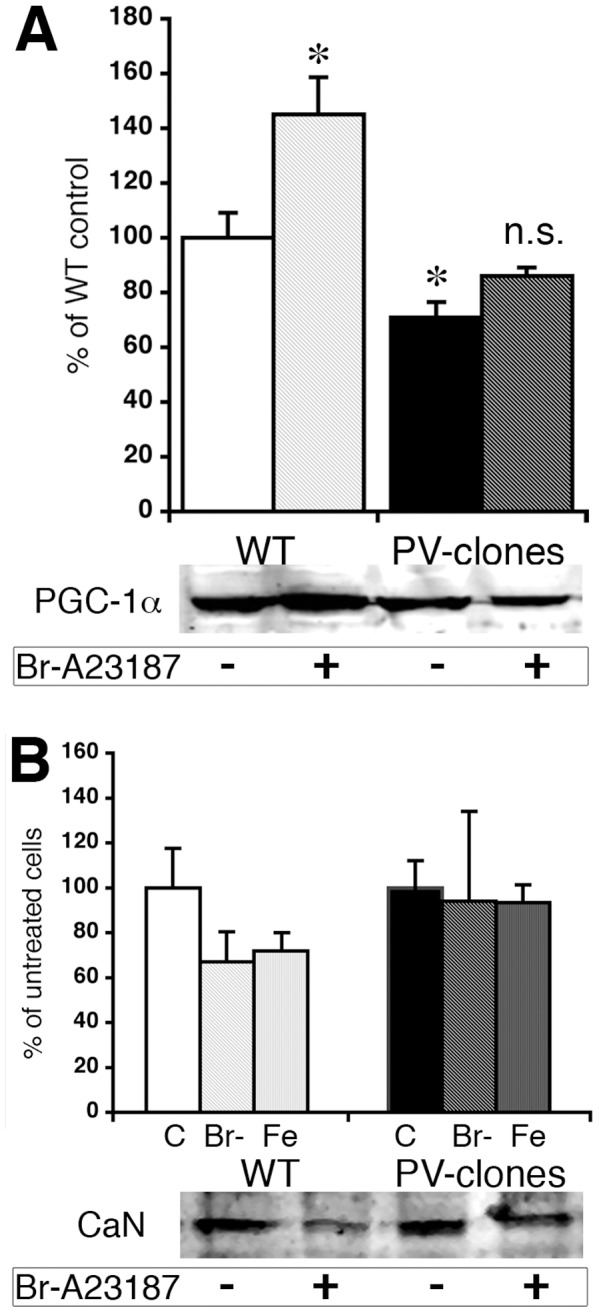
Expression levels of PGC-1α and CaN in C2C12 cells treated with Ca^2+^ ionophores. A) Semi-quantitative analysis for PGC-1α. The immunoreactive band used for the quantification migrated with an apparent M_r_ of ∼90 kDa (calculated M_r_ of PGC-1α is 90,588 Da), at an identical position as PGC-1α from TA extracts (Fig. 7C). Mild ionophore treatment (1 µM; 48 h) led to an increase in PGC-1α in WT C2C12 cells (*; p<0.05). In untreated PV-clones, PGC-1α levels were decreased compared to control C2C12 cells (*; p<0.05); ionophore treatment didn’t additionally affect PGC-1α levels (n.s.). In the lower part representative Western blot signals used for the calculations are shown. B) Analysis of CaN expression, other details as in A). CaN expression levels were determined in untreated cells (C) or after incubation with the Ca^2+^ ionophores Br-A23187 (Br-) or ferutinin (Fe) for 48 h. CaN signals were slightly decreased in WT clones (n.s.) and were unchanged in the PV-clones after ionophore treatment. Lower part: Representative CaN Western blot signals for C2C12 WT and PV-clones ± Br-A23187.

Alterations in Ca^2+^-dependent signaling pathways in fast-twitch muscles of PV−/− mice were proposed to be involved in the increased mitochondria biogenesis [17], including Ca^2+^-dependent kinases and/or phosphatases (see also below). In line with this hypothesis, CaN mRNA levels and CaN activity are increased in PV−/− TA [17]. Also PGC-1α considered to be a master regulator of mitochondria biogenesis is indirectly regulated in a Ca^2+^-dependent manner via an AMPK-SIRT1-PGC-1α signaling pathway [35]. Thus, protein levels of PGC-1α and CaN were investigated in C2C12 with and without PV expression, under basal conditions and after ionophore treatment. Under basal (control) conditions, PGC-1α levels in nuclear extracts were lower in PV-expressing myotubes (−29%±7%; p<0.05) compared with control myotubes (Fig. 5A). Treatment with Br-A23187 for 24 h resulted in a clear increase (45±14%; p<0.01) of PGC-1α in the control (WT) clones, while PGC-1α levels in PV-expressing myotubes were essentially unchanged, despite the increase in [Ca^2+^]_c_ (Fig. 5A). A different situation was observed for CaN. Under basal conditions, CaN levels were similar in control and PV-expressing myotubes and treatment for 48 h with both, Br-A231987 and ferutinin slightly decreased nuclear CaN in control myotubes, but not in PV-clones. Ferutinin, an electrogenic naturally occurring ionophore is considered to be a more physiological tool to induce mitochondrial Ca^2+^ overload than electroneutral ionophores such as Br-A23187 [28]. Therefore, the ionophore-induced elevation in [Ca^2+^]_c_ in PV-clones caused an increase in PV expression (Fig. 1C, D) and decreased mitochondrial volume (Figs. 2 & 3), while the level of nuclear PGC-1α was not significantly changed. In control, PV-devoid myotubes, more resembling “slow-twitch” fibers or fast-twitch fibers from PV−/− mice, ionophore treatment led to an increase in PGC-1α (Fig. 5A) that was also reflected by increased mitochondrial volume (Fig. 3) as observed in PV−/− TA *in vivo*. This was not the case for CaN (Fig. 5B). Ionophore-stimulated WT C2C12 myotubes (thought to mimic physically active PV−/− muscles) had unaltered or even lower CaN levels, unlike increased CaN as in fast-twitch muscle of PV−/− mice. Based on these results, we concentrated more on the PGC-1α pathway.

### Involvement of Sirtuin-1 (SIRT1) in PGC-1α -mediated Regulation of Mitochondrial Volume and PV Expression

**Figure 6:**
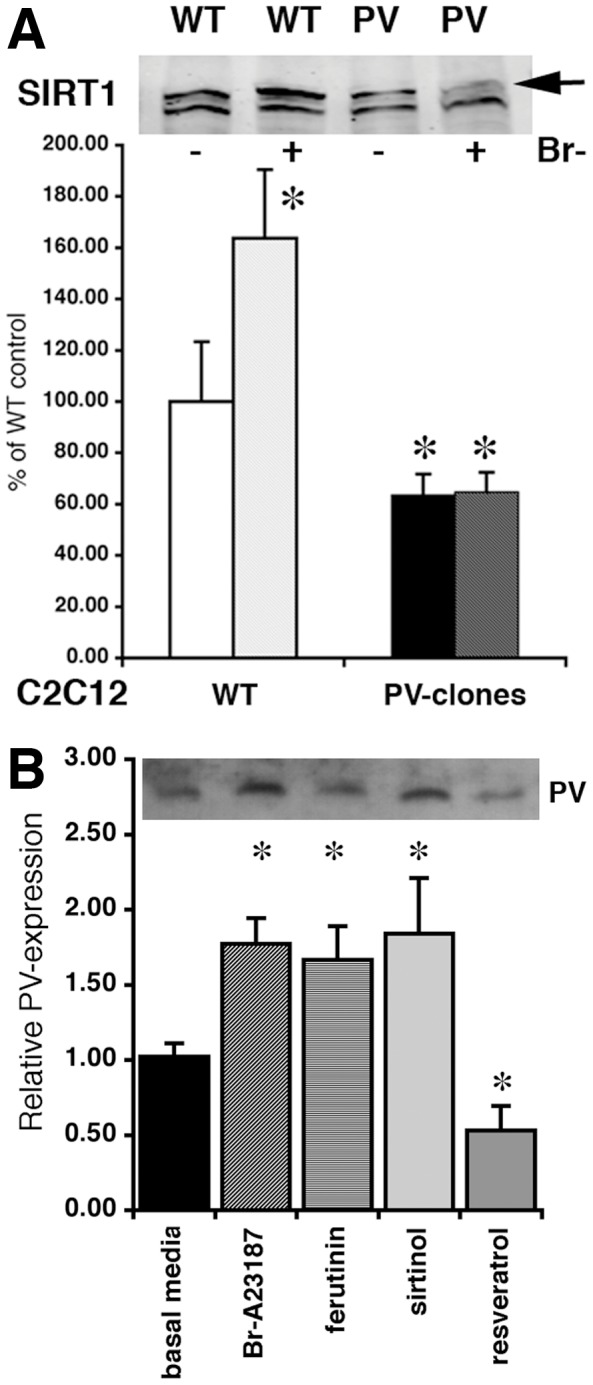
Expression levels of SIRT1 and SIRT1-dependent regulation of PV expression in C2C12 PV-clones. Exposure of WT C2C12 to Br-A23187 for 48 h increased SIRT1 expression (arrow) in WT C2C12 cells exposed to Br-A23187 determined by Western blot analysis; quantitative results are shown in the histographs (*: p<0.05) SIRT1 levels in PV-clones were lower, even under control conditions and ionophore treatment didn’t cause a change in SIRT1 expression in PV-clones (*; p<0.05 compared to WT, basal conditions). Only the upper band (arrow) is the specific band corresponding to the expected size of SIRT1 and was used for the quantitative analysis. B) At 48 h post-treatment PV expression levels in C2C12 PV-clones were increased by the ionophores Br-A2319987 and ferutinin or by blocking of SIRT1 activity by sirtinol. On the contrary, addition of resveratrol activating the SIRT1/PGC-1α signaling axis led to a decrease in PV expression (*, p<0.05 vs. untreated C2C12 PV-clones).

It was hypothesized that the induction of mitochondria biogenesis, i.e. an increase in mitochondrial volume caused by altered Ca^2+^ signaling, e.g. by ionophore-induced elevation in [Ca^2+^]_c_ in C2C12 cells (this study) or by subtle Ca^2+^ signal changes in the TA of PV−/− mice [20] could be mediated through a sirtuin-dependent pathway. Under basal conditions, SIRT1 levels in control, non-transfected C2C12 nuclear extracts from myotubes was approximately 57% higher than in the PV-expressing myotubes (Fig. 6A). In non-transfected myotubes subjected to ionophore treatment for 48 h, the protein level of nuclear SIRT1 was further increased by 64±27% (p<0.05), while no increase was detectable in the PV-expressing myotubes. Thus, the presence of PV likely prevented the ionophore-induced increase in nuclear SIRT1 occurring in the control, PV-devoid myotubes. In order to confirm the assumption that SIRT1 may be linked to the PV/mitochondria balance, the deacetylase activity of SIRT1 was pharmacologically modulated by the (indirect) SIRT1 activator resveratrol and the inhibitor sirtinol. Blocking of SIRT1 activity by sirtinol for 48 h and thus favoring the acetylated, less active form of PGC-1α resulted in an upregulation of PV protein levels to 184% of control (Fig. 6B; p<0.01). A similar upregulation as observed by treatment with Br-A23187 (177%; see also Fig. 1D) or ferutinin (166%) for the same time period. Conversely, PV expression levels were significantly reduced by approximately 50% by increasing the activity of PGC-1α via activation of SIRT1 by resveratrol (Fig. 6B). Collectively, these results indicated that increased PGC-1α e.g. caused by the activation of the AMPK-SIRT1 pathway leads to a down-regulation of PV and concomitantly, an increase in mitochondrial volume.

### Absence of PV also Leads to an Increase in PGC-1α and SIRT1 in TA Muscle of PV−/− Mice *in vivo*

**Figure 7:**
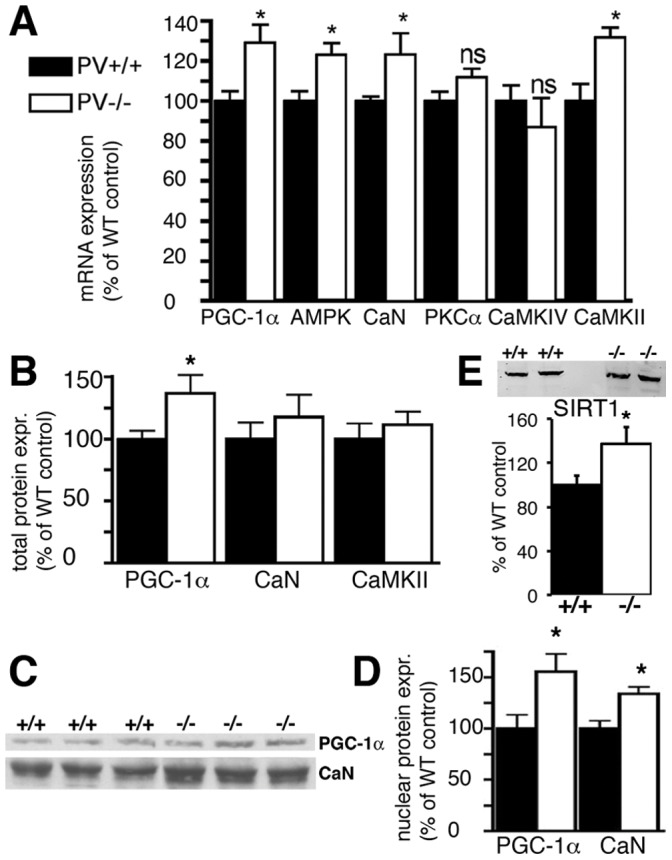
Expression levels of molecules implicated in excitation/transcription coupling and mitochondria biogenesis in mouse skeletal muscle. A) Differences in mRNA levels in *tibialis anterior* (TA) from adult PV+/+ and PV−/− mice determined by RT-PCR. Significant increases were observed for PGC-1α, AMPK, CaN and CaMKII (*; p<0.05). Semi-quantitative Western blot analyses with whole muscle TA protein extracts (B) and TA nuclear extracts from PV+/+ and PV−/− mice (D). Significant increases in PV−/− nuclear PGC-1α and CaN were detected compared to PV+/+ mice (*p<0.05). C) Representative Western blots for PGC-1α and CaN from three PV+/+ and PV−/− mice. Note the stronger signals in extracts from PV−/− mice. E) SIRT1 expression levels in TA from adult mice were increased in PV−/− mice; representative SIRT1 Western blot signals from 2 mice of each genotype (upper panel).

The hallmark of PV−/− fast-twitch muscle is the significantly increased mitochondria volume. Thus, we set out to determine expression levels of Ca^2+^ signaling toolkit components putatively linked to mitochondria biogenesis including kinases and phosphatases. Besides the previously reported increases in mRNA levels of CaN and CaMKII in PV-/- TA (Fig. 7A) and [17], we analyzed other proteins implicated in mitochondria biogenesis, in particular, those regulated (directly or indirectly) in a Ca^2+^-dependent manner. At the level of transcript expression, AMPK and PGC-1α were increased in TA from PV−/− mice, while no differences were seen for CaMKIV and PKCα (Fig. 7A). Western blot analysis from TA whole muscle extracts revealed a small increase in PGC-1α, while the slight increase in CaN and CaMKII did not reach statistical significance (Fig. 7B). For PGC-1α and CaN, showing the largest differences between WT and PV−/− in TA whole muscle extracts, protein levels in nuclear extracts from TA were determined (Fig. 7C, D). Both proteins were significantly enriched in nuclear extracts from PV−/− TA muscle. Finally increased levels of SIRT1 (+37%), the activator of PGC-1α, were detected in nuclear-enriched extracts of PV−/− TA muscle (Fig. 7E). This indicates that the increase in mitochondria volume caused by PV’s absence is mediated by the SIRT1-PGC-1α pathway.

## Discussion

Muscle plasticity is characterized by adaptive changes including changes in muscle fiber type in response to various external stimuli such as physical exercise. Despite uncertainties about the precise signaling mechanisms involved, excitation-transcription (E–T) coupling translates the nerve-evoked electrical activity to a switch in fiber type-specific gene expression [7]. Modifications in Ca^2+^ signaling appear most suitable to translate muscle activity into intracellular signaling pathways. Consistent with this, the regulation of Ca^2+^ signaling toolkit components, the Ca^2+^ homeostasome [36], displays plasticity in order to interpret and convey information in a precise time-dependent and tissue-specific manner. Here an insight is given on the role and regulation of two Ca^2+^-homeostasome components in skeletal muscle: parvalbumin and mitochondria, the latter representing an organelle involved in a multitude of cell functions including an important role in Ca^2+^ signaling, also in skeletal muscle [34]. PV and mitochondria have a dual role, both can act as transient Ca^2+^ sinks and Ca^2+^ sources (“off” and “on” mechanisms [1]) depending on the cellular and temporal context. In this study, PV expression and mitochondria content were compared in two models: *in vitro* using C2C12 muscle cells with or without expression of PV and moreover, *in vivo* comparing WT (PV-expressing) and PV-null mutant (PV−/−) mice. Results obtained in the two models show that PV expression levels and mitochondria volume in skeletal muscle is inversely regulated likely in a bidirectional manner involving the PGC-1α/SIRT1 signaling axis.

Transient changes in [Ca^2+^]_c_ are linked to muscle fiber transition, both from slow-to-fast, and fast-to-slow [37] and a main focus was on the changes in muscle-type specific isoforms including myosin heavy chains. To mimic this situation, the expression of PV was examined in transfected C2C12 cells subjected to Br-A23187 treatment. The ionophore treatment did not give rise to a significant increase of PV mRNA; but PV protein expression levels were clearly augmented by this treatment indicating a post-transcriptional mechanism. The mitochondrial volume was lower in PV-expressing myotubes already at basal conditions, suggesting that Ca^2+^ signaling that occurs in proliferating cells and/or cells subjected to differentiation from myoblasts to myotubes is sufficient to cause the down-regulation of mitochondria. Treatment with ionophore for 48 h had no additional effect on mitochondrial volume, while in PV-devoid (WT) myotubes subjected to the same treatment, mitochondrial volume was significantly upregulated. Mitochondria show a large plasticity with respect to protein composition, morphology and size and are tailored to the metabolic and signaling needs of each cell type and tissue [38]. Whether differences in the kinetics of mitochondrial Ca^2+^ uptake and resistance to K^+^-induced decrease of ψ_m_ between the PV-expressing and PV-devoid C2C12 myotubes is solely the result of increased mitochondrial volume, or is also caused by a selective (up)-regulation of one of the >1000 mitochondrial proteins described in MitoCarta [39], in particular the ones involved in mitochondrial Ca^2+^ handling remains to be investigated. Thus, C2C12 myotubes with or without PV expression may be considered as a valuable model system (besides mouse models with altered PV expression levels [19], [20]) to gain more insight in skeletal muscle plasticity with respect to PV expression and mitochondria volume in the context of Ca^2+^ signaling/homeostasis. Questions that remain to be answered include: (i) what causes the changes in the Ca^2+^ signaling toolkit to adapt to changing demands; (ii) does PV itself possibly act as a local Ca^2+^ sensor? We hypothesize that upon increased muscle activity and associated increased Ca^2+^ signaling activity in PV-expressing muscle fibers an increase in PV expression in order to increase the slow-buffering capacity might be the least demanding choice from an energetic point of view. In fibers not expressing PV (e.g. slow-twitch) an increase in slow-buffering capacity could be mediated by increasing the mitochondrial volume and its associated Ca^2+^ handling capacity. Interestingly, there seems to exist a subtle balance, i.e. an inverse regulation of the two (PV and mitochondria) in all systems analyzed so far.

The molecular mechanisms induced by the presence/absence of PV in either skeletal muscle or C2C12 myotubes were explored with the aim of identifying putative altered signaling pathways. PGC-1α and CaN were increased, at the transcript and protein levels in PV−/− TA. PGC-1α results in C2C12 myotubes were similar, i.e. expression levels were higher in PV-devoid control myotubes than in PV-expressing myotubes, both under basal conditions and more emphatically, after ionophore treatment. For CaN the situation was different. In control clones acutely treated with ionophores (Br-A23187 or ferutinin), CaN levels were decreased, while in the PV-expressing myotubes CaN levels were not affected by either ionophore treatment. The cause for the apparent discrepancy between the *in vivo* and *in vitro* situation with respect to CaN levels may be manifold and include I) chronically higher Ca^2+^-signaling activity in TA vs. acute elevation of [Ca^2+^]_c_ in C2C12 myotubes and II) C2C12 myotube cultures representing only a partial (incomplete) model for an intact muscle fiber. However, the incongruent results from the two models hint that the post-transcriptional regulation of PV expression, at least in C2C12 myotubes, does not likely involve a CaN-dependent pathway. On the other hand, the highly similar pattern of mitochondrial volume and PGC-1α expression levels in both, control and PV-expressing myotubes (compare Fig. 2B, 3B and Fig. 5A), as well as in TA from PV−/− and WT mice (Fig. 7C,D), hinted towards a common inverse/antagonistic regulation of PV and mitochondrial volume via a PGC-1α-dependent pathway. Similarly, in transgenic mice over-expressing PGC-1α in muscle, PV levels in EDL are decreased, while mitochondrial volume is increased [40]. A link between PV and PGC-1α expression also exists in PV-expressing neurons characterized by high expression levels of PGC-1α, albeit the changes are inversed [41]. In PVergic neurons from PGC-1α−/− mice, PV expression levels are significantly reduced, while PGC-1α overexpression in cultured neuroblastoma cells robustly induce PV expression. *Pvalb* transcripts are also reduced in other tissues including retina of PGC-1α−/− mice indicating “PGC-1α is required for proper expression of PV in multiple tissues” [41]. Similarly, PV is an up-regulated target gene of PGC-1α in human white adipocytes, where Ca^2+^ has an anti-lipolytic effect [42]. Thus, a link between PV and PGC-1α regulation is evident, however, the extent and the direction of the regulation (up- or down-regulation) likely depends on several factors, among which the tissue type appears to be crucial.

Sirtuin 1 (SIRT1) is the main deacetylase of PGC-1α that positively regulates genes involved in fatty acid utilization and mitochondrial respiration [11]. Increased levels of SIRT1 up-regulate PGC-1α expression in skeletal muscle (i.e. C2C12 myotubes) and MyoD enhances the binding of the SIRT1/PGC-1α complex to the PGC-1α promoter region. The authors proposed “autoregulatory control of PGC-1α gene transcription seems to be a pivotal mechanism for conferring a transcription-activating response to SIRT1 in skeletal muscle” [12]. Thus, besides the AMPK pathway, also the one involving SIRT1 might converge on PGC-1α mediated signaling to promote skeletal muscle remodeling [43], [44]. Accordingly, our results indicate that SIRT1 might possibly regulate PV expression acting as an upstream signal of PGC-1α. In both models investigated in this study, *in vivo* and in *vitro*, we found SIRT1 expression to be elevated in association with PV-deficiency in TA muscles from PV−/−mice (Fig. 7E) as well as after ionophore-treatment of control (PV-devoid) myotubes (Fig. 6A). Unexpectedly, SIRT1 inhibition by sirtinol increased PV protein levels, while indirectly activating it by resveratrol decreased PV expression in myotubes (Fig. 6B). However, there is still some debate, whether SIRT1 is required in regulating mitochondrial biogenesis. Mice lacking SIRT1 neither show impairment in exercise-induced mitochondrial biogenesis nor in PGC-1α deacetylation [45]. This contrasts previous findings, where it was shown that nuclear SIRT1 activity stimulates exercise-mediated mitochondrial biogenesis in rat and human muscle, possibly via AMPK activation [46]. Future investigations, possibly combining PV-deficient mice with physical exercise may help to resolve these questions.

The SIRT1/PGC-1α-mediated inverse regulation of PV and mitochondria has to be kept in mind when reflecting on the studies of ectopic PV expression in the heart; PV gene (*Pvalb*) transfer leads to an improvement of diastolic function both *in vitro* and *in vivo* models, in normal and diseased conditions [47], [48]. However, the question was raised how extended PV expression in the long run would affect cardiac properties; for this, heart-directed PV expression in transgenic mice was analyzed [49]. Cardiac morphology and systolic function are not affected and relaxation is moderately accelerated. However, decreased SR Ca^2+^ content hints towards adaptation/compensation mechanisms induced by ectopic PV expression also in the heart. Most importantly, based on this study, the long-term effects of PV expression on heart mitochondria biogenesis/function should be investigated; specifically if the therapeutic effects of PV expression will be aimed towards human applications. Focusing on the energy demand of cardiac function, PGC-1α has recently been suggested as a novel therapeutic candidate for metabolic modulation in cardiovascular diseases [50], [51]. Based on these findings, it is hypothesized that cardiomyocytes with alterations in Ca^2+^ signaling might be modified, i.e. PV overexpression might lead to a decrease in heart mitochondrial content or possibly to other alterations of Ca^2+^ signaling components, e.g. linked to the decrease in the SR load [49]. Thus, further mechanistic studies are necessary and the obtained results might have considerable implications for basic muscle physiology, but are expected to also have an impact on clinical concepts aimed to restore muscle (including heart) function.
